# Earliest evidence of dental caries manipulation in the Late Upper Palaeolithic

**DOI:** 10.1038/srep12150

**Published:** 2015-07-16

**Authors:** Gregorio Oxilia, Marco Peresani, Matteo Romandini, Chiara Matteucci, Cynthianne Debono Spiteri, Amanda G. Henry, Dieter Schulz, Will Archer, Jacopo Crezzini, Francesco Boschin, Paolo Boscato, Klervia Jaouen, Tamara Dogandzic, Alberto Broglio, Jacopo Moggi-Cecchi, Luca Fiorenza, Jean-Jacques Hublin, Ottmar Kullmer, Stefano Benazzi

**Affiliations:** 1 Department of Biology, University of Florence, Via del Proconsolo, 12, 50122 Firenze, Italy; 2 Department of Cultural Heritage, University of Bologna, Via degli Ariani 1, 48121 Ravenna, Italy; 3 Sezione di Scienze Preistoriche e Antropologiche, Dipartimento di Studi Umanistici, Corso Ercole I d’Este 32, Università di Ferrara, 44100 Ferrara, Italy; 4 Plant Foods in Hominin Dietary Ecology Research Group, Max Planck Institute for Evolutionary Anthropology, Deutscher Platz 6, 04103 Leipzig, Germany; 5 Institut für Ur- und Frühgeschichte und Archäologie des Mittelaters, Eberhard Karls Universität Tübingen, Schloss Hohentübingen, 72070 Tübingen, Germany; 6 Department of Human Evolution, Max Planck Institute for Evolutionary Anthropology, Deutscher Platz 6, 04103 Leipzig, Germany; 7 Dental Workshop Bensheim, Private Laboratory for Training, Research and Methods, Siegfriedstraße 104, 64646 Heppenheim, Germany; 8 CeSQ, Centro Studi sul Quaternario ONLUS., Via Nuova dell’Ammazzatoio 7, I-52037 Sansepolcro (Arezzo), Italy; 9 Università degli Studi di Siena, Dipartimento di Scienze Fisiche, della Terra e dell’Ambiente, Unità di Ricerca Preistoria e Antropologia, Via Laterina 8, 53100 Siena, Italy; 10 Department of Anatomy and Developmental Biology, Monash University,Melbourne VIC 3800, Australia; 11 Earth Sciences, University of New England, Armidale NSW 2351, Australia; 12 Senckenberg Research Institute, Senckenberganlage 25, 60325 Frankfurt am Main, Germany

## Abstract

Prehistoric dental treatments were extremely rare, and the few documented cases are known from the Neolithic, when the adoption of early farming culture caused an increase of carious lesions. Here we report the earliest evidence of dental caries intervention on a Late Upper Palaeolithic modern human specimen (Villabruna) from a burial in Northern Italy. Using Scanning Electron Microscopy we show the presence of striations deriving from the manipulation of a large occlusal carious cavity of the lower right third molar. The striations have a “V”-shaped transverse section and several parallel micro-scratches at their base, as typically displayed by cutmarks on teeth. Based on *in vitro* experimental replication and a complete functional reconstruction of the Villabruna dental arches, we confirm that the identified striations and the associated extensive enamel chipping on the mesial wall of the cavity were produced ante-mortem by pointed flint tools during scratching and levering activities. The Villabruna specimen is therefore the oldest known evidence of dental caries intervention, suggesting at least some knowledge of disease treatment well before the Neolithic. This study suggests that primitive forms of carious treatment in human evolution entail an adaptation of the well-known toothpicking for levering and scratching rather than drilling practices.

Dental caries are a major oral health problem in modern human societies^1^, representing one of the most common chronic dental diseases around the world. The need to treat carious teeth was well-known during historical times as well. To improve pain relief, medieval treatments were based on either humoral theory using herbal remedies or anatomical principles^2^,^3^. Ancient Greeks and mainly Romans were acquainted with caries removal by drilling and cleaning the infected cavity^4^,^5^, and Egyptian texts confirm this practice was established at least in the fifth millennia BP^6^.

The most ancient evidence of dentistry dates back to the Neolithic period, probably associated with the increase in carbohydrate-rich diets [some bacteria^7^ such as *Streptococcus mutans*, convert fermentable carbohydrates to form acids; an increase in acidity might favour the demineralisation of the dental tissues^1^] typical of agricultural societies^8^ when compared with the more varied diet of hunter-gatherers^9^,^10^. Indeed, beeswax dental filling was discovered in ca. 6,500 calibrated years before present (cal yr BP) human tooth from Slovenia^11^, while tooth perforations from a bow drill, presumably to remove decayed tissues, were observed in ca. 9,000 cal yr BP molars from a Neolithic graveyard in Pakistan^12^.

Before the Neolithic, rudimentary forms of oral hygiene were represented by the use of toothpicks (flexible or inflexible probes probably made of bone and/or wood)^13^, potentially to remove food particles between teeth, leaving characteristic interproximal grooves (in the mesial and distal surface of the teeth, but not in the occlusal surface) that are bucco-lingually elongated^14^. This practice is documented from the beginning of the genus *Homo* and is extremely common among Neandertals and Palaeolithic modern humans^13^,^14^. However, because during the Palaeolithic toothpicking is not associated with carious lesions^15^, it is suggested to be an applied measure to alleviate painful gums or simply a habitual idiopathic activity^13^,^14^,^15^. Though toothpick-use was common, Coppa and colleagues^12^ emphasized that unambiguous evidence of true dental treatment (i.e., attempts to remove carious lesions) was only known from the Early Neolithic. Indeed, Lukas and Pastor^16^ categorized toothpicks among the Neolithic individuals at Mehrgarh (Pakistan) as habitual and occupational grooves, and only during the Chalcolithic these grooves can be related to therapeutic purposes.

Here we analyse a lower right third molar (RM_3_) (Fig. 1) of the Late Upper Palaeolithic specimen known as Villabruna showing clear evidence of dental caries manipulation. The specimen is a young male individual (ca. 25 years old) that was recovered in 1988 from the Epigravettian deposit of Riparo Villabruna (Sovramonte—Belluno, Italy), and was directly dated to around 14,160–13,820 cal yr BP^17^,^18^ (Supplementary Information; Supplementary Fig. S1).

## Results

**Figure 1:**
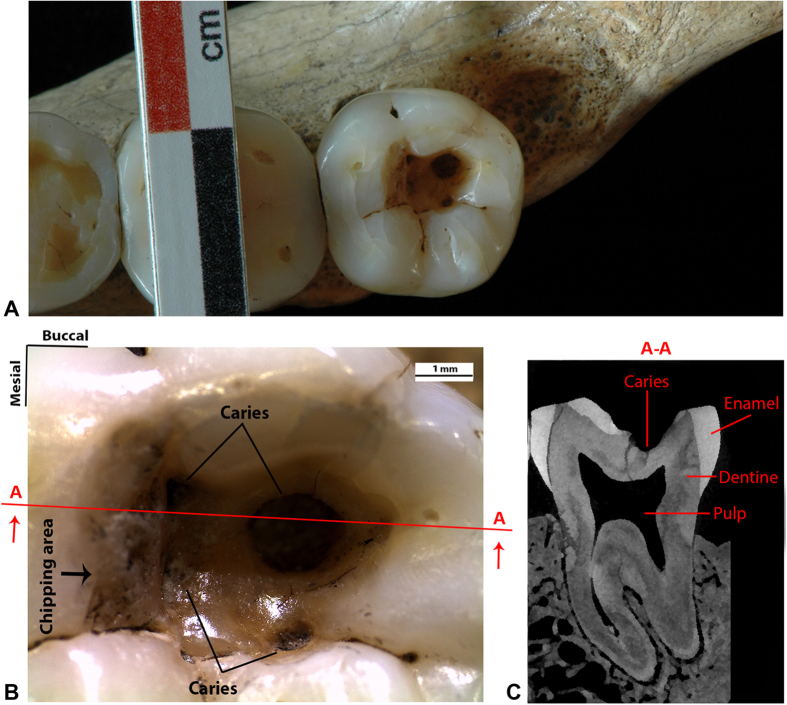
The lower right third molar (RM_3_) of the Late Upper Palaeolithic specimen known as Villabruna. (**A**) Occlusal view of the RM_3_. (**B**) Detailed view of the large occlusal cavity with the four carious lesions and the chipping area on the mesial wall. Section A-A is directed mesio-distally, passing through the larger carious lesion. (**C**) MicroCT slice of the Villabruna RM_3_ in correspondence with section A-A.

The RM_3_ retains a large occlusal cavity (mesio-distal = 5.84 mm; bucco-lingual = 3.33 mm), with a polished internal surface and extensive enamel chipping on the steep mesial wall (Fig. 1A,B) (the other teeth are lacking from caries, except for a tiny hole—incipient caries—in the lingual wall of the RM^3^). The cavity, which is located at the level of the hypoconid and hypoconulid cusps, is sub-squared on the lingual and mesial sides but rounded on the buccal and distal sides. Within the cavity four caries (characterised by demineralised, dark dental tissues) are present^19^. These include three small and shallow pits found in the mesio-buccal and disto-lingual side and a large lesion in the disto-buccal quadrant (mesio-distal = 1.8 mm; bucco-lingual = 1.6 mm; height = 0.96 mm) that penetrates into the dentine producing an empty circular hollow without invading the pulp chamber (Fig. 1C).

**Figure 2:**
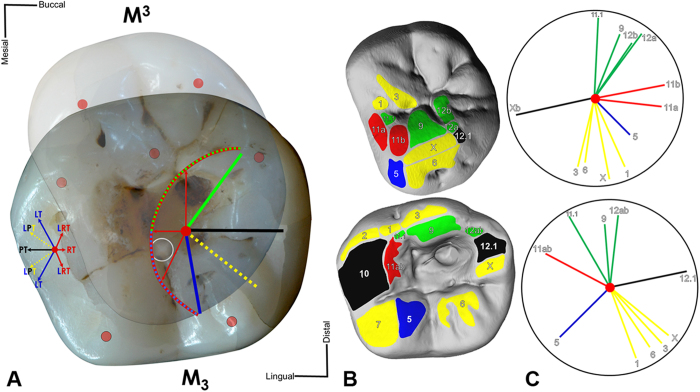
Occlusal relationship between the Villabruna’s RM_3_ and RM^3^. (**A**) Maximum intercuspation between antagonistic crowns. M^3^ transparent and mirrored for occlusal view on M_3_. Red point, cusp tips; grey circle, central fossa center M_3_. The dental occlusal compass (left) designates general directions of movements in mandibular symphysis out of maximum intercuspation. The dental occlusal compass (right) indicates directions of the M^3^ protocone tip (red center point). Protrusion (black); lateroprotrusion (yellow); laterotrusion (blue); retrusions (red); mediotrusion (green). (**B**) Wear facet pattern labeled and color-coded. (**C**) Individual occlusal compass results showing spatial orientation of each wear facet.

A functional reconstruction of the dental arches, derived from the information preserved in the dental macrowear pattern (Supplementary Fig. S2), indicates that the main cusp of the antagonistic molar (the protocone of the RM^3^) is responsible for the majority of the wear facets produced on the RM_3_ (Fig. 2). Corresponding wear facets on the antagonistic M^3^ are located near the cavity, but none of them extend into it. Specifically, the enamel chippings observed in the uppermost mesial margin of the cavity are partially rounded and polished due to wear (facet 11; Fig. 2B), confirming they were produced ante-mortem. A number of chippings deeper in the mesial wall are not affected by wear and possess sharp edges. The surface of the unworn chipped area indicates that fracturing has developed through repeated interactions. Occlusal Fingerprint Analysis (OFA) shows that the protocone touches neither the floor nor the deeper mesial wall of the cavity during occlusal movements (Supplementary Information; Supplementary Fig. S3; Supplementary Videos 1,2), suggesting that the chippings were not produced during masticatory activities.

**Figure 3:**
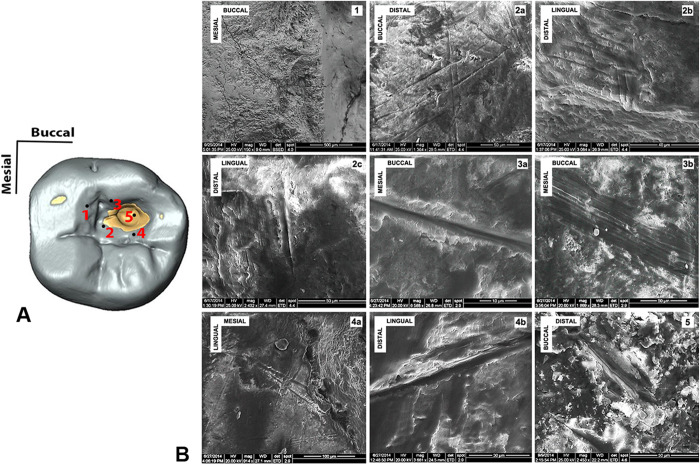
Scanning Electron Microscopy (SEM) images of the striations observed within the carious cavity of the Villabruna RM_3_. (**A**) Occlusal view of the RM_3_ digital model, with underlined some of the areas where striations were observed. (**B**) The SEM images: 1, the chipping area; 2a-b-c, the mesial area; 3a-b, the buccal wall of the cavity; 4a-b, the lingual wall of the cavity; 5, inside the large carious lesion.

Scanning Electron Microscopy (SEM) analysis (Quanta Inspect S, FEI Company Hillsboro, USA) of the internal cavity surface reveals striations (Fig. 3). These are clearly different from typical dental microwear (microscopic scratches), which is produced by attritional contact between lower and upper teeth and by the action of abrasive particles during masticatory activities^20^. These striations are well documented within the cavity, and occur even on the bottom of the larger disto-buccal carious lesion (Fig. 3). However, they gradually disappear towards the occlusal surface probably as a consequence of tooth wear, thus confirming (along with the wear facet covering the mesial chippings) their ante-mortem formation.

**Figure 4:**
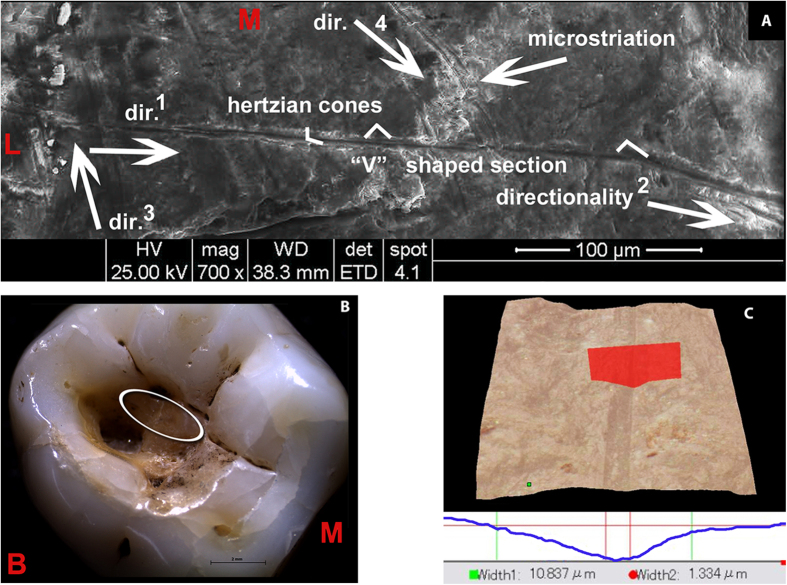
Morphological description of the striations observed in the Villabruna RM_3_. (**A**) SEM images with morphological and directionality striation features (the numbers indicate the sequence of the gestures). (**B**) Stereo microscopical image of Villabruna RM_3_ with magnification of the cavity and of the region (ellipse) containing the striations described in this figure (region 2 and 4 in Fig. 3). (**C**) Example of 3D rendering and cross-section of the striation observed in the Villabruna tooth cavity (area 2 in Fig. 3). B, buccal; L, lingual; M, mesial.

Viewed microscopically and in cross-section, these striations exhibit similar morphological features to cutmarks on bone^21^, i.e., Hertzian cones, grooves with a “V” shaped transverse section and microstriation at the bottom, sharply defined, with a high apex, steep sides, narrow cross-sections and well-defined parallel ancillary ridging (Figs 3 and 4; Supplementary Fig. S4).

According to the inferred directionality of the traces, the employed flint tools passed through mesio-buccal > distal, mesial > distal and lingual > disto-buccal axes (Supplementary Fig. S5A,B,C). The study of the orientation of individual traces within the cavity (Supplementary Fig. S5D) indicates a variety of gestures and movements associated with the slicing of the tool edges in different directions. However, it is possible to distinguish two main groups of lines (i.e., lingual > buccal and mesial > distal; Supplementary Fig. S5D, yellow) potentially produced during back and forward semi-circular levering and scratching movements.

Experimental tests carried out on the enamel of three recently extracted M_3_s using experimental wood, bone and *microgravettes* Epigravettian stone points confirm that the striations observed in the Villabruna specimen were caused by microlithic points (Supplementary Information; Supplementary Table S1). The wood point did not leave any mark on the enamel surface (Supplementary Fig. S6). The bone point produced only extremely faint scratches (Supplementary Fig. S7), whereas the Epigravettian microlithic point produced striations that are identical to those observed in the Villabruna specimen (Supplementary Fig. S8). This was confirmed by further experiments on six carious human teeth using microlithic points on exposed dentine (Supplementary Information; Supplementary Fig. S9; Supplementary Table S2). The experimental striations also resemble those described by other authors in relation to the action of cutting pieces of meat, held between the anterior teeth, with a stone tool^22^,^23^. However, the striations on the Villabruna RM_3_ appear only within the cavity on the occlusal surface, and would therefore not have been exposed during meat-cutting.

Gas Chromatography-Mass Spectrometry (GC-MS) was used to characterise i) residue adhering to the inner cavity of the RM_3_, ii) remnants of what appears to be a mass of organic material integrated within a carbonate concretion buried near the left iliac crest which could possibly be the material used to treat RM_3_, and iii) traces of residue adhering to the ilium, potentially originating from the same organic material found in the carbonate concretion (Supplementary Information; Supplementary Fig. S1)^17^,^18^. A negligible result was obtained for the residue in the inner cavity of the RM_3_, as well as from all the samples tested except for one, namely the residue adhering to the ilium (Supplementary Table S3). The profile obtained for this result is suggestive of a natural wax^24^ (Supplementary Fig. S10), which could have been locally sourced. A beeswax origin could also be tentatively made, based on the advanced decay of the characteristic alkane profile and the identification of a disaccharide moiety. However, it was not possible to obtain direct evidence for possible therapeutic-palliative medication of the RM_3_.

The presence of dental caries in the Villabruna specimen may suggest a diet rich in carbohydrate intake. We tested whether there were plant or other micro-remains preserved within the dental cavity, using methods slightly modified from those published previously^25^, which were designed to control for potential sources of contamination. The few starches recovered show similar morphology to those found in the control samples from the packing material (Supplementary Table S4) and are therefore likely to be the result of contamination and cannot be considered indicative of diet.

## Discussion

The substantially smaller extension of the demineralised (decayed) tissue when compared with the extensive size of the cavity itself, and the presence of chippings and striations even in the most inaccessible areas of the larger carious pit, strongly suggest intentional (albeit partial) removal of the carious infected/decayed tissue. The Villabruna specimen represents therefore the oldest archaeological evidence of operative manual intervention on a pathological condition (caries), as indicated by the striations on the bottom of the carious pit (Fig.3; Supplementary Fig. S4C), potentially to remove the caries and/or to re-establish antagonistic tooth function by removing food particles entrapped within the cavity. This evidence predates the ca. 9,000 cal yr BP Neolithic dental drilling documented in Pakistan^12^, as well as the earliest undisputed evidence of cranial surgery, currently represented by the ca. 9,000-7,000 cal yr BP trephination from Vasilevka II, Ukraine^26^ and Ensisheim, France^27^ [cranial trephination predating the Mesolithic, as the examples suggested by Dastugue^28^,^29^, are highly dubious^26^,^27^]. Therefore, we suggest that the earliest dental caries manipulation entails an adaptation of the toothpicking technique from simple rubbing actions between interproximal teeth using probes made on bone/wood, to scratching/levering activities within the carious lesion using microlithic points.

Recent studies show that dietary changes towards a more carbohydrate-rich diet (e.g., large exploitation of grains and starches) may have occurred well before the Neolithic, predating the origin of agriculture by ca. 10,000 years^30^,^31^, if not 20,000 years^32^. Though it is undeniable that the frequency of dental caries increased with the advent of agriculture^9^,^10^, some regions may have experienced a dietary shift during the mid-Late Upper Palaeolithic, as suggested by a greater incidence of carious lesions (rarely observed in fossil hominins)^33^ in some modern human populations^34^. The rise in caries incidence, coupled with appropriate lithic technology during the Late Upper Palaeolithic may have created an optimal context within which to adapt the habitual use of a toothpick (made of wood/bone) towards a rudimentary dental intervention using microlithic tools. Like other Late Upper Palaeolithic cultures, the Epigravettian was characterised by widespread production of backed artifacts made from bladelets, generally used as insets for weaponry^35^. Specifically, the microgravettes were elongated and strong points designed for use as hafted hunting projectiles (Supplementary Fig. S9A), but their small size and hardness were well suited both to enter into small carious cavities and to remove the demineralised but resistant bacterially infected enamel and dentine tissues by levering and scratching (Supplementary Fig. S5A,B,C).

Therefore, the earliest incipient dentistry entails levering and scratching but not drilling practices, as observed later, during the Neolithic and in modern dentistry.

## Methods

### Micro-CT scan

High-resolution micro-CT images of the Villabruna upper and lower dentition were obtained with a BIR Actis5 microtomographic system (Max Planck Institute for Evolutionary Anthropology, Leipzig, Germany) using the following scan parameters: 130 kV, 100 μA, with 0.50 mm Brass filter. Volume data were reconstructed using isometric voxel length of 30 μm. The micro-CT images of the teeth were virtually segmented using a semiautomatic threshold-based approach in Avizo 7 (Visualization Sciences Group Inc.) both to reconstruct a complete 3D virtual model of the Villabruna dentition and to evaluate the extension of the carious lesion in the RM_3_ (Fig. 1C).

### Reconstruction of physiological occlusal relationship

The functional reconstruction of the Villabruna dentition follows indications provided by Benazzi *et al.*^36^ and Kullmer *et al.*^37^. In detail, the upper and lower dentition of the Villabruna specimen was reproduced with high-resolution epoxy and dental stone casts^38^. Moreover, digital surface data of the dentition was acquired with a white light 3D digitisation system (smartSCAN^3D^, Breuckmann GmbH, Germany), with an average resolutions of ~65 μm.

The casts of the upper and lower third molars were used to draw two-dimensional maps of all complementary wear facet pairs on the occlusal surface after their identification with a binocular (Leitz MZ12). In addition, the facets were interactively marked on the virtual models using PolyWorks® 12.0 software (InnovMetric Software Inc., Canada)^39^.

The facets were labelled applying the numbering system of Maier and Schneck^39^, and colour-coding in the facet maps follows^39^,^40^,^41^. The application of the dental occlusal compass determines relative occlusal movements for each individual wear facet pair. The point of maximum intercuspation (centric occlusion) marks the start point of directions of movements for the standardised colour-coding in OFA^39^. Blue coloured facets indicate occlusal contacts during latero- and lateroretrusive movements, yellow specifies lateroprotrusion facets, green shows medioretrusive movements, red and black indicate retrusion and protrusion, respectively (Fig. 2). The facet maps are used to identify directions of occlusal movements^41^,^42^, and support setup of the condyle boxes of the dental articulator.

For physiological repositioning of the teeth we aligned casts of each tooth crown in a dental articulator system (PROTAR, KaVo Dental GmbH, Germany). With a dental articulator system it is possible to reproduce natural occlusal movements, while macroscopically observe the contact situations^36^,^37^. The epoxy cast specimens were positioned in the articulator after taking its lower jaw dimensions (general geometry, condylar axis position, occlusal and the mid-sagittal plane) from a 3D-print of the complete lower jaw (data from micro-CT).

After positioning of the mandibular resin dentition in the articulator, the maxilla epoxy cast was positioned with the best-fit occlusal relationship possible. Once the initial position is setup, the epoxy casts are replaced with dental stone copies on a wax basis. A slight distortion in the original specimen prevents a proper occlusion. Therefore we used dental stone copies, which can be easily cut at the interproximal planes that each crown can be repositioned independently. Both arches were mounted with dental gypsum between duett-plates and montage-plates (Baumann Dental GmbH) in the articulator. All crowns were then removed from the arches bases.

The upper and lower right M3s of the right and left sides were repositioned first. Based on their occlusal fingerprint (wear facet pattern) they provide important occlusal precision for matching the antagonistic pairs. When the third molar pairs are positioned in maximum intercuspation, we set up the articulator condyle boxes to constrain possible articulator movements for the individual occlusal simulation. Subsequently the antagonistic occlusal pairs can be restored in their dental arches in the same way. The articulator allows testing of the occlusal position of each repositioned tooth pair to ensure consistency of functional movements throughout the tooth rows in accordance with the colour-coded occlusal compass. The anterior dentition of Villabruna was reconstructed last, because it does not show any contact in maximum intercuspation of the dental arches.

### Virtual Occlusal Fingerprint Analysis (OFA)

Virtual Occlusal Fingerprint Analysis was applied to evaluate the physiological occlusal movements and crown contacts. The upper and lower jaw digital models were aligned with a virtual model generated from a surface scan from the physical reconstruction of the dental arches in maximum intercuspation (Supplementary Fig. S2), using a best-fit algorithm in IMInspect module of PolyWorks® 12.0.

We verified the kinematics of the occlusal movements (i.e., the pathway of incursive and excursive movements) applying the “Occlusal Fingerprint Analyser” (OFA) software. The OFA software is a virtual tool developed at the Senckenberg Research Institute in Frankfurt (Germany) to detect relief guided dental collisions of antagonistic tooth crowns^43^,^44^,^45^. The OFA software records the occlusal pathways and sequential surface contacts derived from collision detection, deflection and breakfree algorithms (Supplementary Fig. S3; Supplementary Videos 1,2).

### Experimental replication of striations

#### Test 1

Experimental scratching/levering activities were carried out on the enamel surface of three recently extracted lower M_3_s to test three kinds of point tools (Supplementary Table S1): wood point (Supplementary Fig. S6C), bone point (Supplementary Fig. S7C) and microlithic point (Supplementary Fig. S8C). These tools were produced by Matteo Romandini e Rossella Duches (University of Ferrara). The wood point was produced on *Larix decidua*, as coniferous trees dominated the landscape during the period of the burial. The bone point was obtained from the diaphysis of a large size ungulate, and is comparable to bone points found in the burial kit (Supplementary Fig. S1). The microlithic point was made by direct retouching of bladelets extracted from red and grey flint cores, comparable to those exploited by the Epigravettian settlers at Riparo Villabruna.

The same force was applied during the tests. The breakage of the point defined the end of the experiment (Supplementary Table S1).

#### Test 2

Experimental tests were carried out using microgravette Epigravettian points on six medieval carious human molars (Supplementary Table S2) collected from the Department of Cultural Heritage (University of Bologna, Italy). Different forces were applied in relation to dentine exposure, and several parameters were evaluated, such as the type of tool used (tool shape efficacy), actions, directions, inclinations and duration of treatment (Supplementary Table S2).

### Analysis of the cross-sectional geometry

The Villabruna RM_3_ and the six archaeological human molars used for the experimental tests were analysed using a Hirox Digital Microscope KH-7700 with an MXG-10C body, OL-140II and OL-700II lenses and an AD-10S Directional Lighting Adapter. This portable instrument, housed at the University of Siena, provides a 3D composite image through the overlapping of a series of pictures taken at different focus levels. It enables us to observe the cross section of grooves and to collect metrical parameters^46^,^47^, as recently shown for the study of archaeological cutmarks and interproximal grooves on human teeth^13^,^47^,^48^. The following metrical parameters were collected: DC (depth of cut), BT (breadth at the top), BF (breadth at the floor) and RTF (ratio between the breadth at the top and the breadth at the floor of cut).

Three striations within the Villabruna RM_3_ cavity were analysed. Two are located in the region 2 and one in the region 5 (Fig. 3). The striations are shallow (DC is less than 4 μm) and narrow (BT between 2.5 and 17.5 μm), and cross sections are V-shaped (Supplementary Fig. S4A,B,C). This characteristic is quantified by the high value of ratio between breadth at the top and breadth at the floor of grooves (RTF comprised between 6.3 and 8.3).

Experimental grooves inflicted by the use of Epigravettian points on exposed dentine are V-shaped, with RTF ranging from 5.2 and 13.1 (n = 6) (Supplementary Fig. S9), resembling those observed in the Villabruna RM_3_.

### Gas Chromatography-Mass Spectrometry (GC-MS)

Beeswax has already been identified in ancient therapeutic dental practices^11^, and could potentially have been used in the case of the Villabruna individual. Another related possibility which has antibacterial and antifungal properties is propolis, a sticky material that honeybees collect from living plants, mix with wax and use to construct and repair their hives^49^. The chemical composition of both beeswax and propolis are known^49^,^50^, and characterisation can be carried out using organic residue analysis (ORA).

Sampling was carried out by CDS at the Max Planck Institute for Evolutionary Anthropology, Leipzig. Scrapings of the residue adhering to the outer and inner surfaces of the ilium were obtained for testing using a sterilised scalpel. A sample was taken from the organic material integrated within a carbonate concretion buried in close proximity to the left iliac crest, and the surrounding soil was tested as a control. The material adhered to the cavity in the molar proved difficult to sample. To avoid damaging the inner surface of the tooth, repeated washings with small quantities of an organic solvent (dichloromethane:methanol, 2:1, *v:v*) were taken using a sterilised glass Pasteur pipette to directly dissolve organic compounds present in the residue. Supplementary Table S3 reports the sampling details.

#### Solvent Extraction

All solvents used were HPLC grade solvents (Roth), and the standard purity was ≥99% (Sigma-Aldrich). Glassware was sterilised before use and a method blank was included to monitor laboratory contamination. Isotopically labelled C_18:0_ was used as an internal standard for quantification purposes.

Prior to extraction, 10 μg of isotopically labelled C_18:0_ internal standard were added to all the samples. To each sample, 2 mL of dichloromethane:methanol (2:1; *v:v*) solution were added. The samples were shaken and sonicated for 15 minutes, and then centrifuged (3500 rpm, 10 minutes, room temperature). The solvent containing the extracted lipid was pipetted into screw capped test tubes and the extraction was repeated twice more, combining the lipid extracts. The solvent was then evaporated to dryness under a gentle stream of nitrogen and mild heating (30 °C) to obtain the total lipid extract (TLE). Each sample was rehydrated using 120 μL of hexane and then partitioned [1:1]. The solvent was evaporated, and both parts of the samples were stored at −20 °C pending further analysis. One part of each sample was derivatised (silylated) and analysed, the other stored.

#### Saponification

Potential ‘unbound’ lipid fractions in samples VIL01, VIL02, VIL03 and VIL04 were targeted by saponification. To each sample, 1 mL of 0.5 M methanolic sodium hydroxide solution made up in methanol:water (9:1, *v:v*) was added. The samples were shaken and vortexed, and then heated (90 minutes, 70 °C). The samples were allowed to cool and centrifuged (4000 rpm, 10 minutes, room temperature). The supernatant was pipetted into screw-capped test tubes. The neutral fraction was extracted three times using 1 mL of hexane into small glass vials. The aqueous fraction was acidified to a pH 3 using *c.*0.4 mL of  6M hydrochloric acid. The acid fraction was extracted three times into small vials, using 1 mL of hexane. Both the acid and neutral fractions were evaporated to dryness using mild heating (30 °C) and a gentle stream of nitrogen. The neutral fraction was silylated prior to GC-MS analysis, while the acid fraction was methylated before silylation and GC-MS analysis.

#### Methylation

200 μL of Boron Triflouride (14% Methanol) were added to each of the samples, which were then heated for 1 hour at 70 °C. The reaction was quenched with 2 drops of double distilled water, and allowed to cool. Methylated lipids were extracted three times using 2 mL hexane. Samples were evaporated to dryness using mild heating (30 °C) and a gentle stream of nitrogen, then rehydrate using 120 μL of hexane and partitioned [1:1]. The solvent was evaporated and samples store at −20 °C pending further analysis. One part of each sample was silylated prior to GC-MS analysis.

#### Silylation

30 μL of pyridine were added to the dried samples at room temperature, followed by 55 μL of MSTFA (*N*-Methyl-*N*-trifluoroacetamide). The samples were agitated for 30 minutes at 37 °C, then centrifuged to remove any remaining drops on the snap caps, and transferred to autosampler vials containing micro inserts.

#### Gas-Chromatography Mass-Spectrometry (GC-MS) Analysis

GC-MS analysis was carried out on an Agilent 6890 Gas Chromatograph coupled with a Quadrupole Mass Spectrometer (MS) (Agilent, Germany), equipped with an Agilent 7683 series auto sampler (Agilent, Germany). A Hewlett Packard 5973 Mass Selective Detector (MSD) was used for GC-MS analysis. The GC was fitted with a 30 m DB-5MS (5% phenyl methyl siloxane) Agilent column, with a 0.25 mm internal diameter and a film thickness of 0.25 μm. The samples were injected in splitless mode at 300 °C. Helium was used as the carrier gas, with a flow rate of 1 mL min^−1^. The oven was programmed at 50 °C for 2 minutes, then ramped at 10 °C per minute to 325 °C and held for 15 minutes. The MS was operated in Electron Impact mode (EI; 70 eV), at a full scan range of *m/z* 50 to 550, with a scan time of 3s per scan. Data acquisition was carried out using Data Analysis Version 3.3 (Bruker Daltonics) data system. Data analysis was performed using MSD ChemStation Version D.00.01.

### Plant microremain analysis

The Villabruna remains were brought to the archaeological material laboratory in the Max Planck Institute for Evolutionary Anthropology, where they were sampled by AGH. The caries on the lower molar was sampled for possible plant microremains by adding a small volume of double distilled water (~50 μl) to the cavity using an adjustable volume pipet with a plastic disposable tip, agitating the surface by pumping the water in and out of the pipet, and finally transferring all of the water to a microcentrifuge tube. Later 1 ml of ddH2O was added, and the tube vortexed for 15 sec, and centrifuged for 5 min at 3 krpm. 950 μl of supernatant was removed and the pellet resuspended in the remaining 50 μl, 10 μl of which was mounted on a slide, with 10 μl 25% glycerin.

We also collected several kinds of control samples, including samples of the containers in which the fossil material was stored, to look for contamination from the post-excavation curation. We took samples from the bubble wrap and stuffing in the box in which the mandible was stored, as well as the stuffing from the skull box and small fragments from the bottom of the skull box. These controls were sampled by holding them with forceps over a 15 ml tube, and washing them with a stream of ddH2O which was collected in the tube. The tube then centrifuged, then all but ~50 μl removed, and 10 μl of this remainder mounted and examined. Finally, when a batch of samples was mounted for a day’s worth of microscopy, we prepared a blank slide, which contained only 10 μl dH2O and 10 μl 25% glycerin.

We performed regular cleaning and testing procedures to assess possible lab contamination. The laminar flow hood and the surrounding bench areas were cleaned once a week with hot tap water and starch-free soap, followed by a wipe with 5% bleach, and a final tap water wipe rinse. Since the results of Crowther and colleagues^51^, we now recommend using NaOH instead of bleach. Every two weeks, the laminar flow hood and the bench work area were tested for contaminants by wiping the entire surface with a wet towel, rinsing the towel into a 50 ml tube, centrifuging this tube, pipetting off the supernatant and mounting the remainder on a slide. Records were kept of the contaminant load before and after cleaning, with photographs and written descriptions to allow comparison to the archaeological material. All of our reagents and mounting material were changed once a month, and the water and glycerin containers were tested once every two months for contaminants. In addition to the weekly cleaning, the work areas were cleaned immediately prior to sampling with soap and a water rinse.

## Additional Information

**How to cite this article**: Oxilia, G. *et al.* Earliest evidence of dental caries manipulation in the Late Upper Palaeolithic. *Sci. Rep.* **5**, 12150; doi: 10.1038/srep12150 (2015).

## Supplementary Material

Supplementary Information

Supplementary Video 1

Supplementary Video 2
