# Supplementary material for: Earliest evidence of dental caries manipulation in the Late Upper Palaeolithic
Source: Sci Rep. 2015 Jul 16;5:12150. doi: 10.1038/srep12150 (PMC4504065; doi:10.1038/srep12150)
Supplement: Supplementary Information [file srep12150-s1.pdf]

SUPPLEMENTARY INFORMATION

Earliest evidence of dental caries manipulation in the Late Upper Palaeolithic

Gregorio Oxilia, Marco Peresani, Matteo Romandini, Chiara Matteucci, Cynthianne Debono Spiteri, Amanda G. Henry, Dieter Schulz, Will Archer, Jacopo Crezzini, Francesco Boschin, Paolo Boscato, Klervia Jaouen, Tamara Dogandzic, Alberto Broglio, Jacopo Moggi-Cecchi, Luca Fiorenza, Jean-Jacques Hublin, Ottmar Kullmer & Stefano Benazzi

The Villabruna burial

The burial of Riparo Villabruna (Supplementary Fig. S1) was discovered in the interiot of the small shelter named Riparo Villabruna A (Sovramonte – Belluno, Italy). The burial was exposed by a cut made for the purpose of widening a road. Within this process both the lower limbs of the burial were severed at the distal femoral shafts[1]. The subject[2] was an adult male, who was buried outstretched within a grave 30-40 cm deep. The skull was facing towards the individuals left hand side, and was in close proximity to the shelter wall. A container, probably a bag, was placed on the left forearm. Within the container were six objects. These included two refitted fragments of a bone point engraved with two series of notches, a backed knife, a blade made on flint, a core made on flint, a siltstone pebble "retoucher" and a lump of unidentified material with attached carbonate concretions. The blade and core, the siltstone pebble as well as part of both the radius and the iliac crest show differential traces of weathering and surface corrosion.

After the body had been placed in the grave, it was covered with large stones collected on the Cismon e Rosna stream beds, few hundred meters from the shelter. The cobbles were further painted with red ocher[1].

Following the removal of the stones during archaeological excavation, the skeleton was delicately recovered using aspirators, soft brushes and distilled water to remove the finer sedimentary particles. A thin carbonate concretion lens covered the skull surface, and was

manually removed during excavation. The teeth emerged clean and unscathed, and no mechanical intervention was required to clean them.
Radiocarbon dating both on charcoal from the pit sediment (R-2023: 12,040±150 yr BP)[1], and directly of the skull (KIA-27004: 12,140±70)[2] place the burial in the interval 14,400-13,800 cal yr BP (CalPal Calibration Program).

OFA results

The results show that the protocone of the upper right third molar (RM^3) makes contact with the uppermost margin of the mesial wall of the cavity during an orthal incursive movement. . The protocone contact – manifesting in a protrusive movement through the cavity - thereby produced mesio-distally elongated wear facets along the lingual and buccal margins of the cavity (Supplementary Video 1). The movement of the protocone produces a deeply worn and tub-shaped enamel basin along the distal margin of the cavity (Supplementary Fig. S3). An OFA sequence performed with the full dental arch reconstruction produced similar results, which suggests that the cavity was present for a substantial period within the life-time of the individual (Supplementary Video 2).

Experimental replication of striations

First test

Information about the three recently extracted molars, point tool (wood, bone and microgravette Epigravettian point) used, action, direction, inclination and duration of treatment are reported in Supplementary Table S1.
Ind-1 (RM_3): After 10 seconds the bone point broke and its length was reduced of ca. 1 mm (Supplementary Fig. S6).
Ind-2 (LM_3): After 5 seconds the pressure exerted on the hafted backed point immediately caused the micro-fracture of the tool. After 15 seconds chippings on the enamel were observed; after ca. 1'00'' minute the tool was still intact (Supplementary Fig. S7).
Ind-3 (LM_3): After 5 seconds the point broke, and after 28 seconds the tool was considered inefficient (Supplementary Fig. S8).

Second test

Information about the six (carious) medieval human tooth classes, tool (microgravette Epigravettian points) used, action, direction, inclination and duration of treatment are reported in Supplementary Table S2 (see also Supplementary Fig. S9).

Vald-1 (RM_3): After 5 minutes, part of the enamel was removed reaching and exposing the primary dentine tissue. After almost 9 minutes, the tool was still intact.

Vald-2 (RM_3): After 1.26 minutes the first signs of flint breakage appeared. At 2.10 minutes abrasive action of the flint tool produced several striations on the enamel. After 4.00 minutes the enamel was scraped.

Guid-1 (RM_2): After 1.12 minutes of mesio-distal movements, fractures on the flint tool were observed. The changing of movements (i.e., helicoidal lever and twisting) was more efficient, producing several small enamel chippings.

Guid-2 (RM_3): After a few seconds the flint point broke and was consequently reduced in length (ca. 3mm), probably due to the pressure produced through the hafted backed point.

Guid-3 (RM^2): The pressure exerted on the hafted backed point immediately caused the fracture of the tool. Moreover, tiny fragments of flint fell into the carious cavity.

Guid-4 (LM_2): The dominant movement recorded (inverse and direct clockwise force) produced several striations on the exposed dentine. After 2 minutes, chippings appeared on the edge of the tool.

Gas-Chromatography Mass-Spectrometry (GC-MS) results

Very small quantities of lipids were obtained for all samples tested. GC-MS characterization was dominated by non-diagnostic fatty acids, precluding a secure interpretation (Supplementary Table S3). Analysis of the organic material integrated within the carbonate concretion, as well as of the soil control sample revealed a very similar profile, suggestive of a similar origin. A negligible lipid profile with no evidence for diagnostic biomarkers was obtained for the residue adhering to the inner walls of the tooth cavity. The lipid profile obtained for the organic residue deposited on the outer and inner surfaces of the ilium differed from the associated mass of material (Supplementary Fig. S10). It comprised a range of saturated and monounsaturated fatty acids, cholesterol possibly arising from endogenous skeletal lipids ([3], and a suite of alkanes with 24 to 35

carbon atoms. The alkane profile showed no odd or even preference, and none of the alkanes identified dominated the series.

References

1. Aimar, A. *et al.* Les Abris Villabruna dans la Vallèe du Cismon. *Preistoria Alpina* **28**, 227- 254 (1992).
2. Vercellotti, G., Alciati, G., Richards, M. & Formicola, V. The Late Upper Paleolithic skeleton Villabruna 1 (Italy): A source of data on biology and behavior of a 14.000 year-old hunter. *J Anthropol Sci* **86**, 143-163 (2008).
3. Evershed, R.P., Turner-Walker, G., Hedges, R.E.M., Tuross, N. & Leyden, A. Preliminary results for the analysis of lipids in ancient bone. *J Archaeol Sci* **22**, 277-290 (1995).

Supplementary Figures

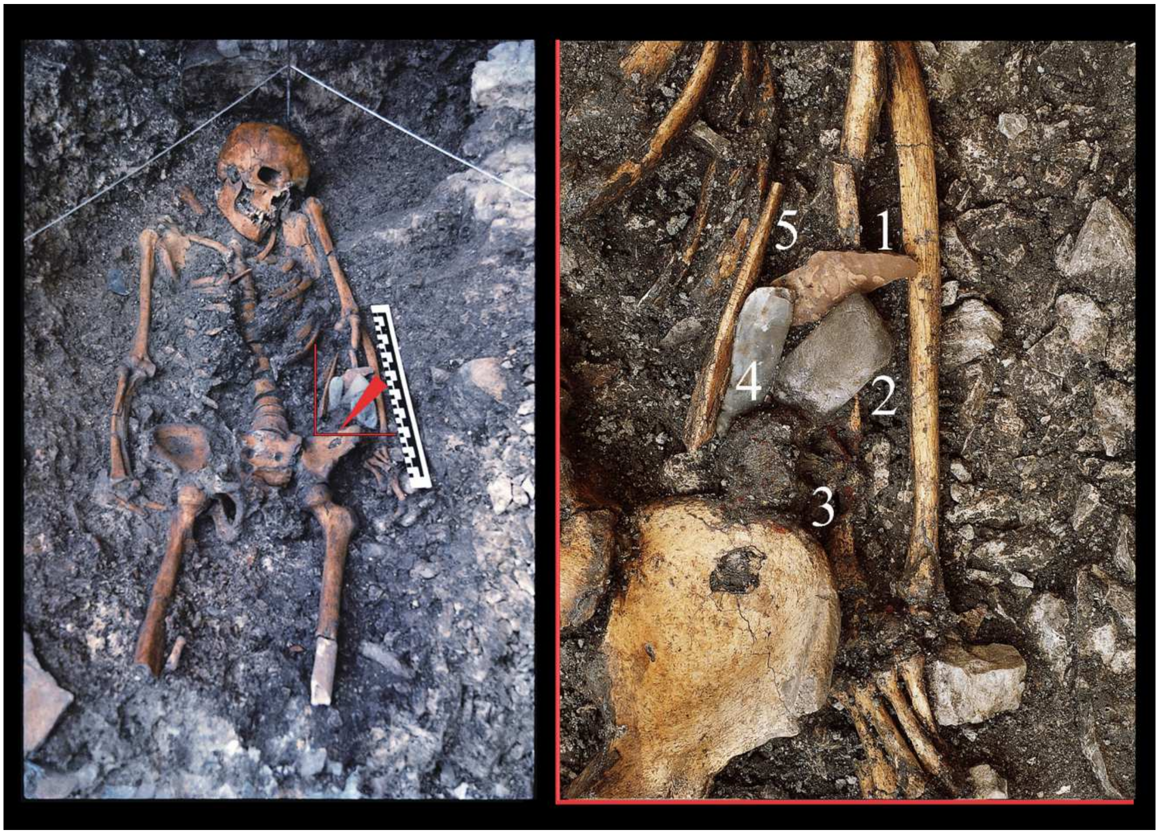

Supplementary Figure S1. The Late Upper Palaeolithic burial Villabruna. Above the left radius, the following items are visible, 12 o'clock clockwise: 1, backed knife; 2, siltstone pebble; 3, remnants of the nodule of unidentified material placed near the left iliac crest; 4, blade; 5, the refitted bone point. The red arrow in the left image points towards the area where residues adhering to the ilium were found (photo by A. Broglio).

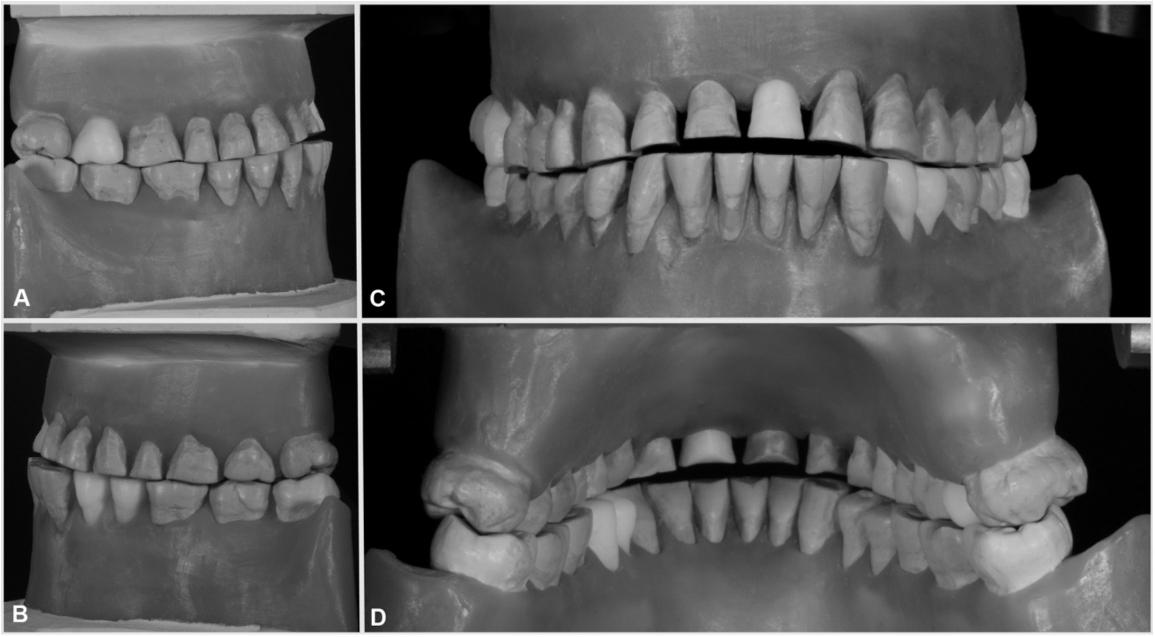

Supplementary Figure S2. Physical reconstruction of the Villabruna dental arches, derived from occlusal wear pattern analysis. (A) Right side. (B) Left side. (C) Frontal (buccal) view. (D) Posterior (lingual) view.

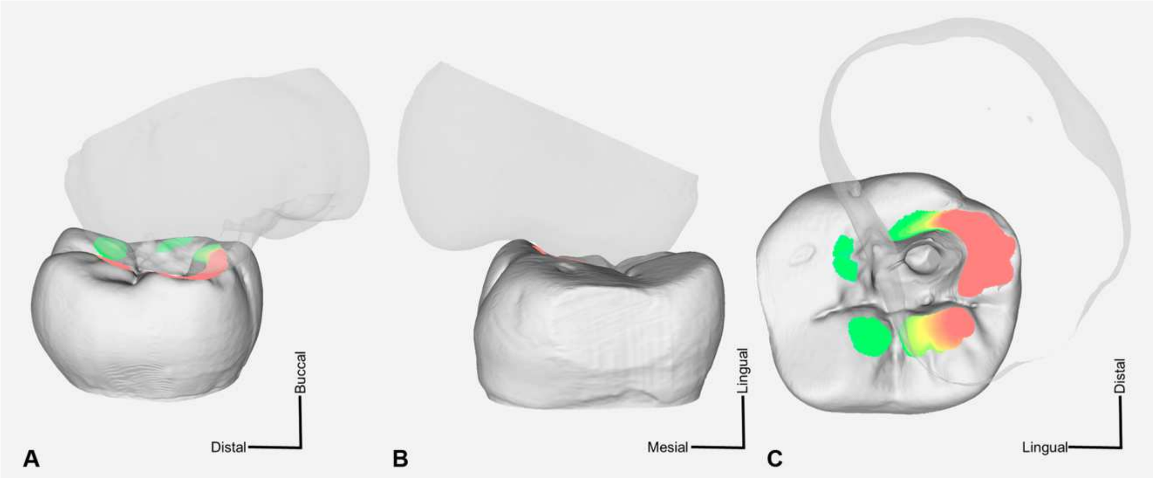

Supplementary Figure S3. OFA results. Snapshots from Occlusal Fingerprint Analyser software (OFA) showing sequential colour gradient of antagonistic occlusal collisions visualized on the M_3 crown from first to last contacts (green to red) during the powerstroke movements. (A) Distal view. (B) Mesial view. (C) Occlusal view. M^3 crown transparent.

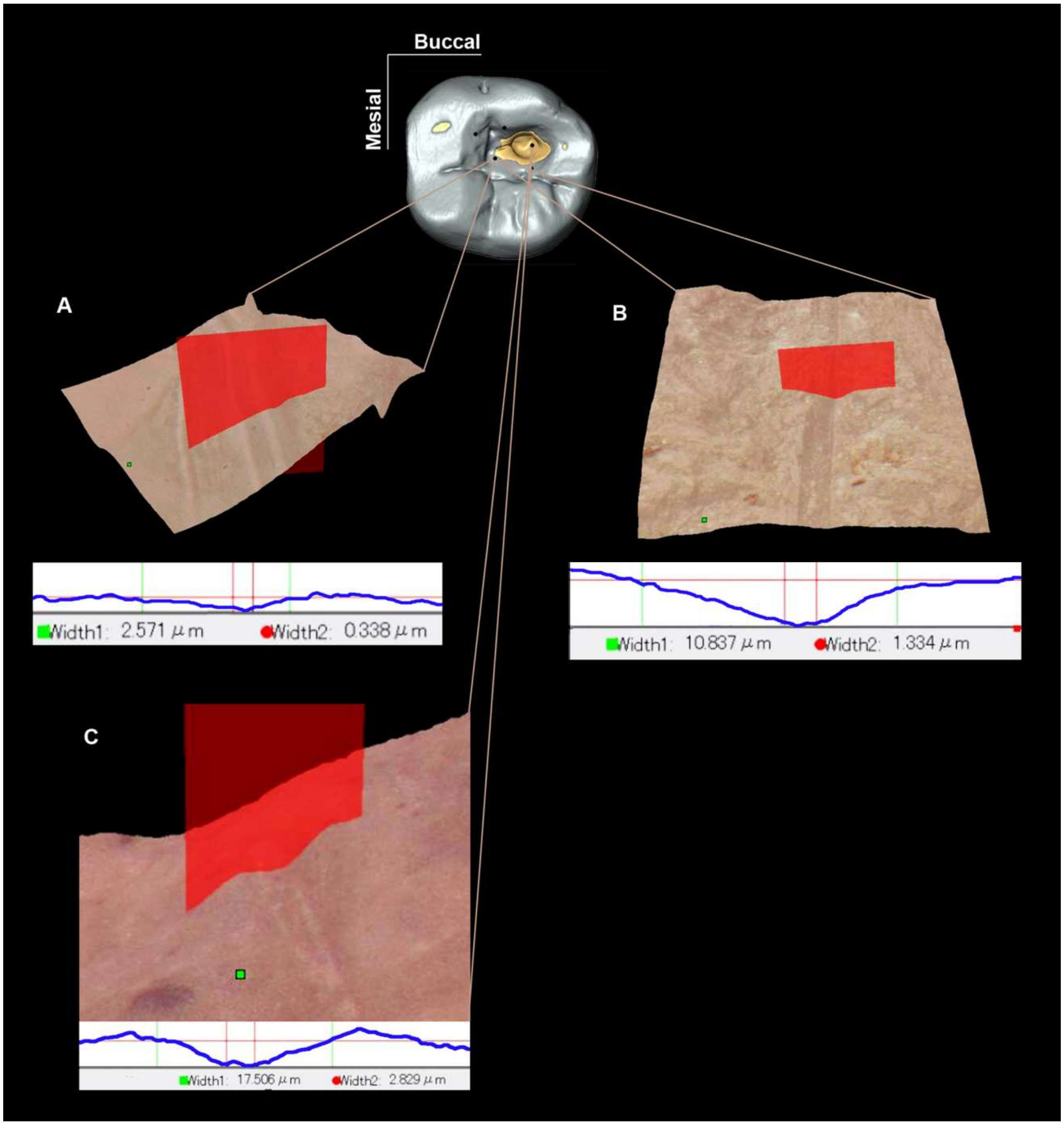

Supplementary Figure S4. 3D rendering and cross-sections of the striations observed in the RM_3 cavity of the Villabruna burial. (A, B) Area 2 in Fig. 3. (C) Area 5 in Fig. 3. The striations are shallow, narrow, and the cross sections are V-shaped. This characteristic is reflected in the high value for the ratio between breadth at the top, and breadth at the floor of the grooves. These data fit very well within the expected variability of marks produced using a flint tool, and resemble experimental flint tool striations represented in Fig. S5.

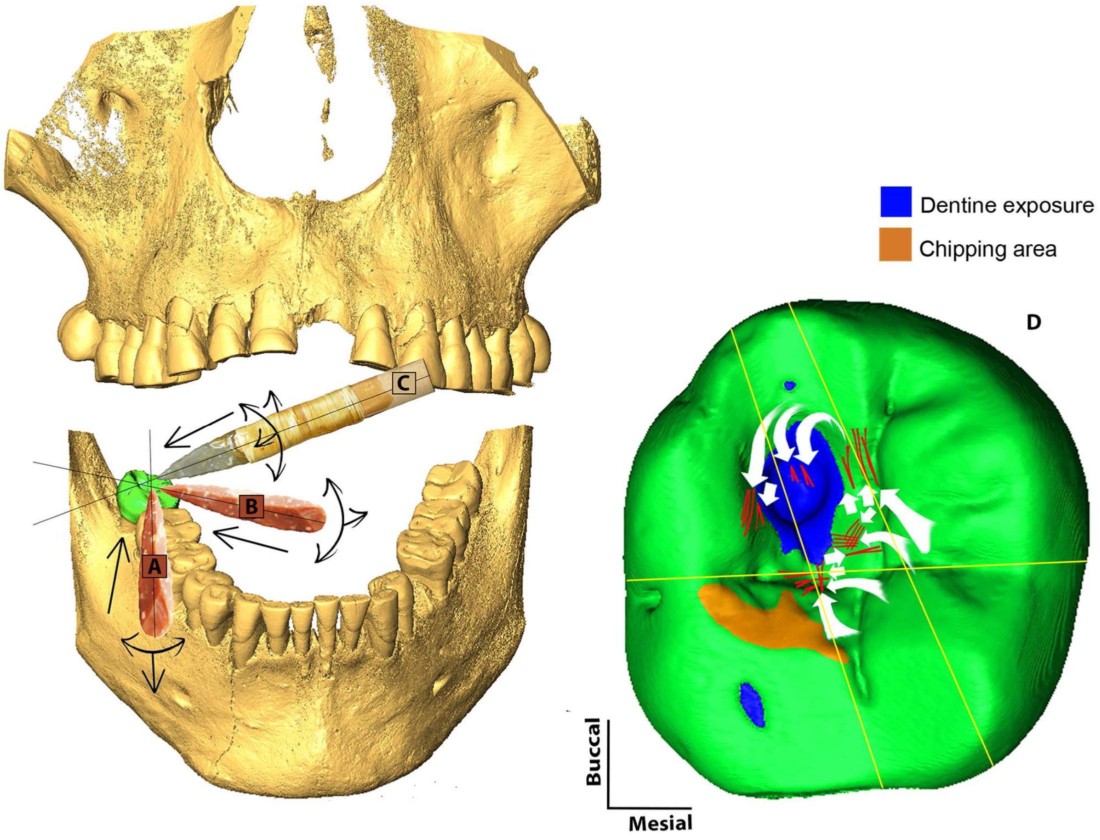

Supplementary Figure S5. Orientation and location of the striations observed in the RM$_3$ cavity of Villabruna. Direction of flint tool action. (A) Mesio-buccal > distal. (B) Mesial > distal. (C) Lingual > bucco-distal. (D) The RM$_3$ of Villabruna with highlighted the position and orientation (white arrows) of the striations (in red). The orientation of individual traces within the cavity indicates a great variety of gestures and movements linked to the running of the siliceous edges in different directions. It is possible to distinguish two main groups of direction lines (yellow) in the entrance-exit of the tool employed.

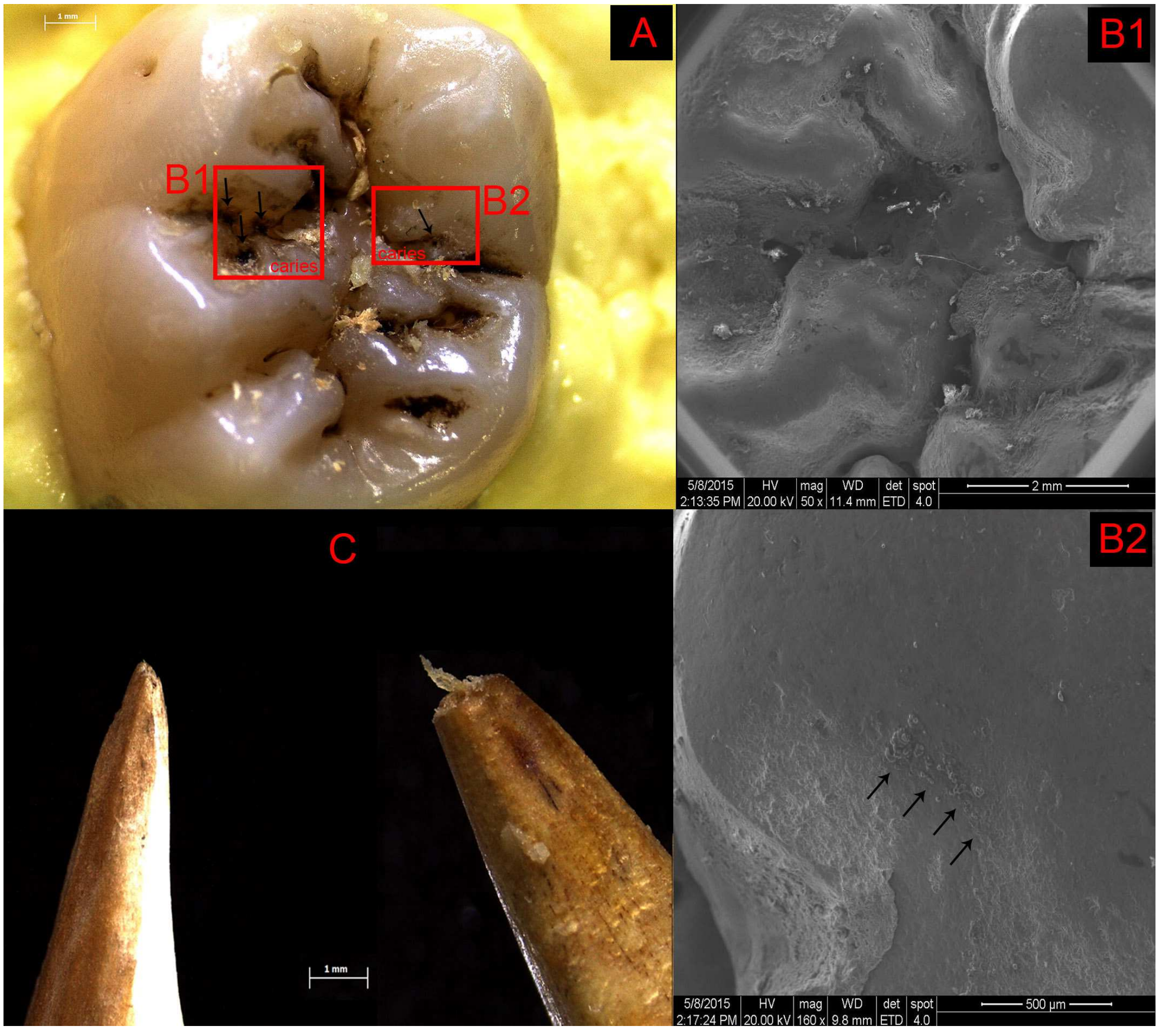

Supplementary Figure S6. Experimental test on the enamel of a recently extracted modern human molar using a wood point tool. (A) Occlusal view of a modern human LM_3 (Ind-3); the squares identified the areas were the wood point was tested. (B1 and B2) SEM images within the areas delimited by the squares shown in A; arrows in B2 point towards wood debris. (C) The wood point before (to the left) and after (to the right) the experimental test.

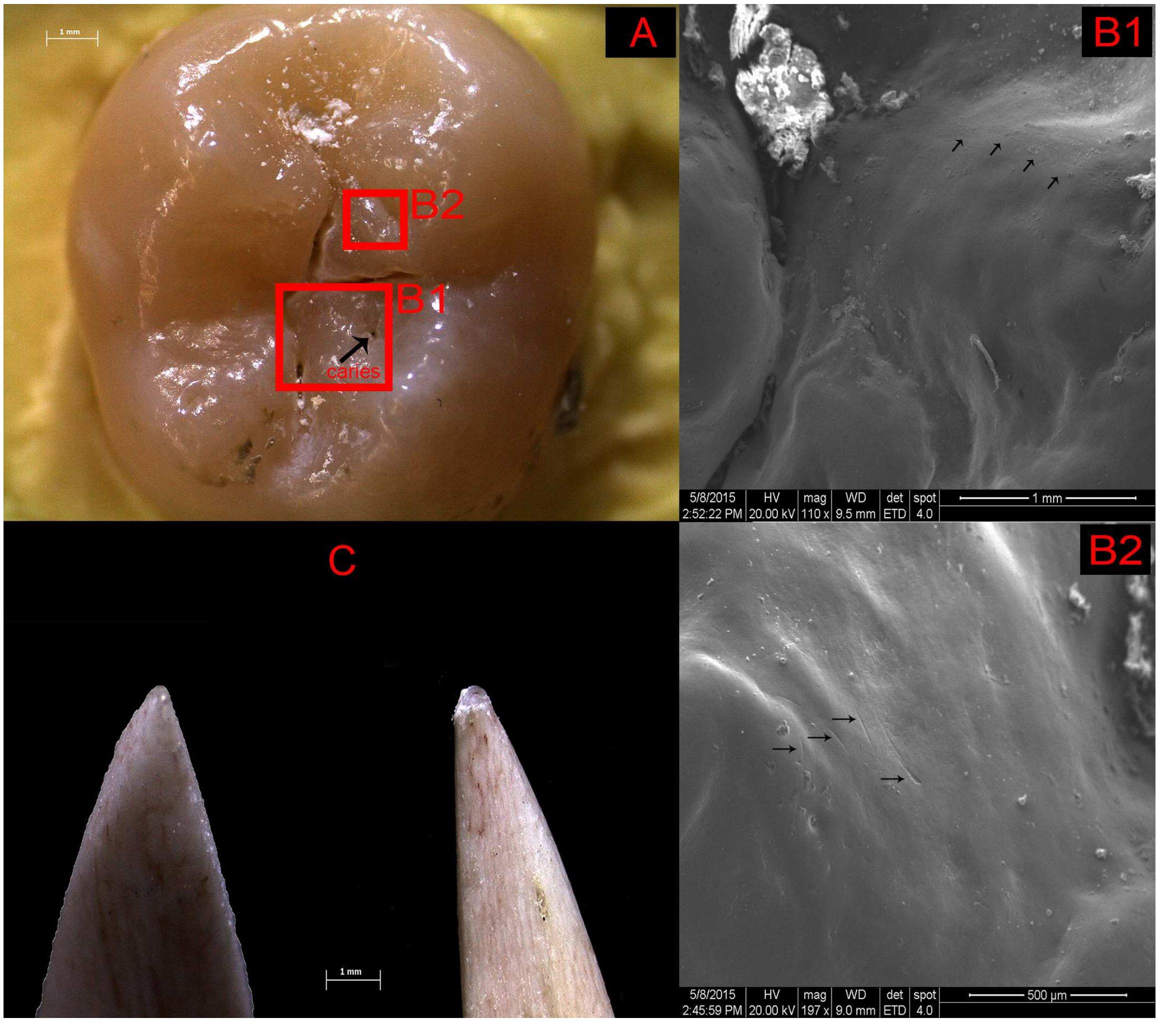

Supplementary Figure S7. Experimental test on the enamel of a recently extracted modern human molar using a bone point tool. (A) Occlusal view of a modern human LM_3 (Ind-1); the squares identified the areas were the bone point was tested. (B1 and B2) SEM images within the areas delimited by the squares shown in A; arrows point towards micro-scratches produced by the bone tool. (C) The bone point before (to the left) and after (to the right) the experimental test.

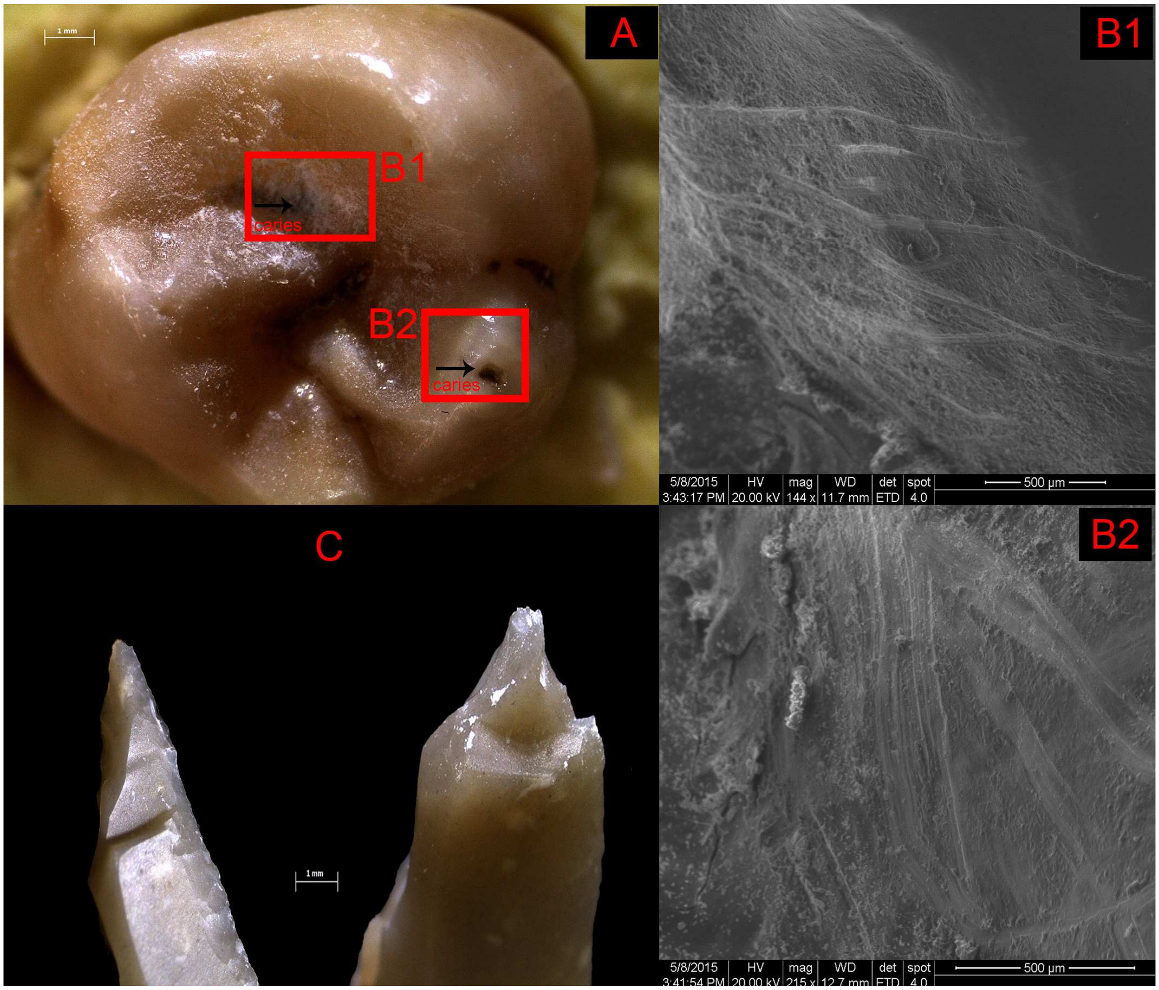

Supplementary Figure S8. Experimental test on the enamel of a recently extracted modern human molar using a microgravette Epigravettian point. (A) Occlusal view of a modern human LM_3 (Ind-2); the squares identified the areas were the microlithic point was tested. (B1 and B2) SEM images within the areas delimited by the squares shown in A; arrows point towards the extensive striations produced by the microlithic point. (C) The microlithic point before (to the left) and after (to the right) the experimental test.

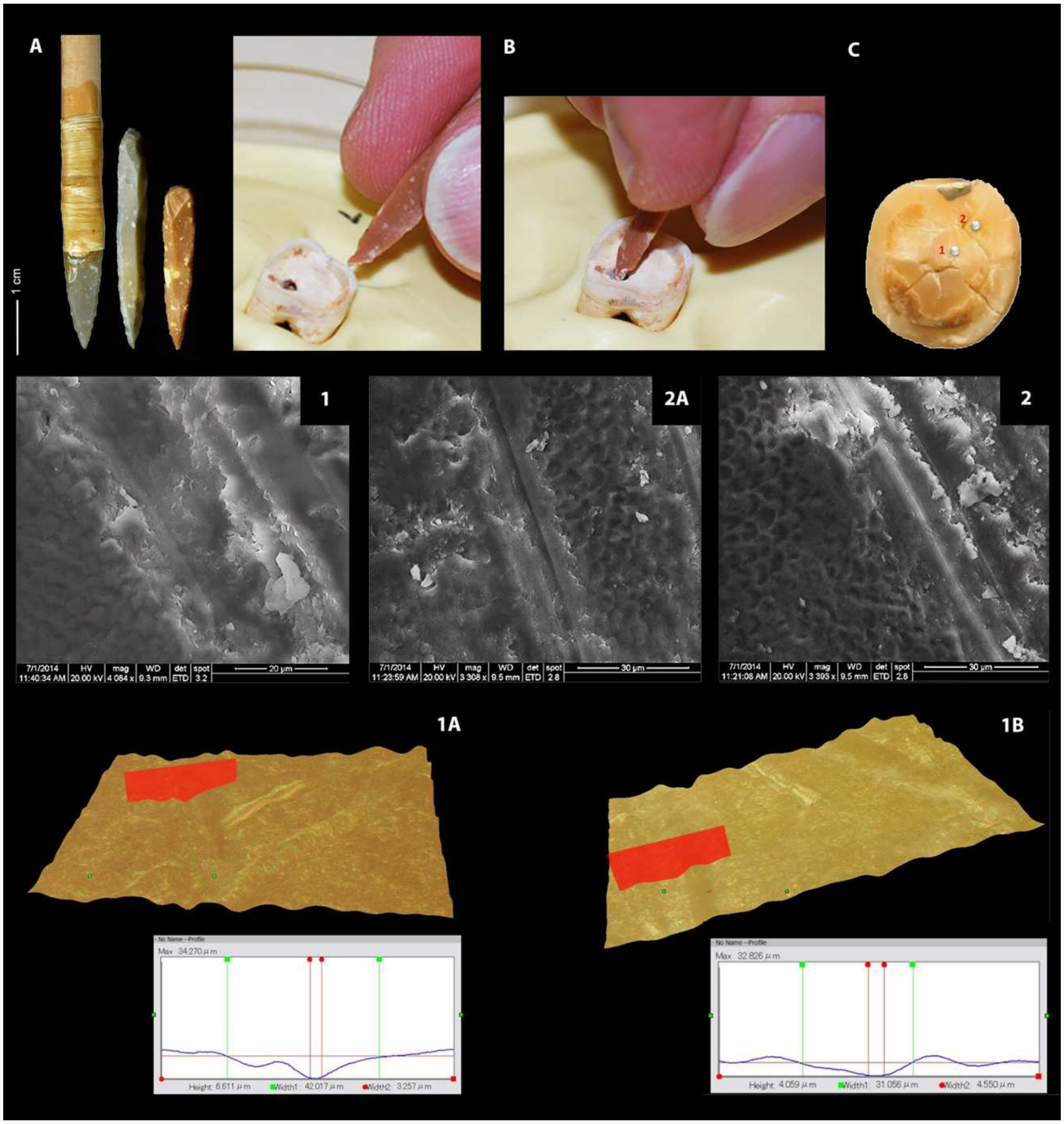

Supplementary Figure S9. Experimental test on modern human molars, SEM and 3D rendering cross-sections analyses. (A) Experimental late Epigravettian backed points made on flint comparable to those used by the Villabruna individual; to the left, hafted backed point used for the lingual > bucco-distal experimental scratching (it corresponds to Supplementary Fig. S4C). (B) Late Epigravettian backed point directly gripped and used on a modern human molar (collected from a bioarchaeological sample at the Department of Cultural Heritage, University of Bologna) which had been set in rubber for this purpose. (C) Occlusal view of a modern human tooth with the location of the main flint tool scratching produced on the exposed dentine (area 1 and 2). For this

tooth, 1, 2a, 2 (middle of the figure) show detailed SEM images of the striations related to area 1 and 2, respectively. On the bottom, 1a and 1b represent 3D rendering and cross-sections of the striation observed in area 1: groove 1a is V-shaped and shows a visible shoulder striation; groove 1b is _/-shaped; its asymmetry can be related to the inclination of the tool.

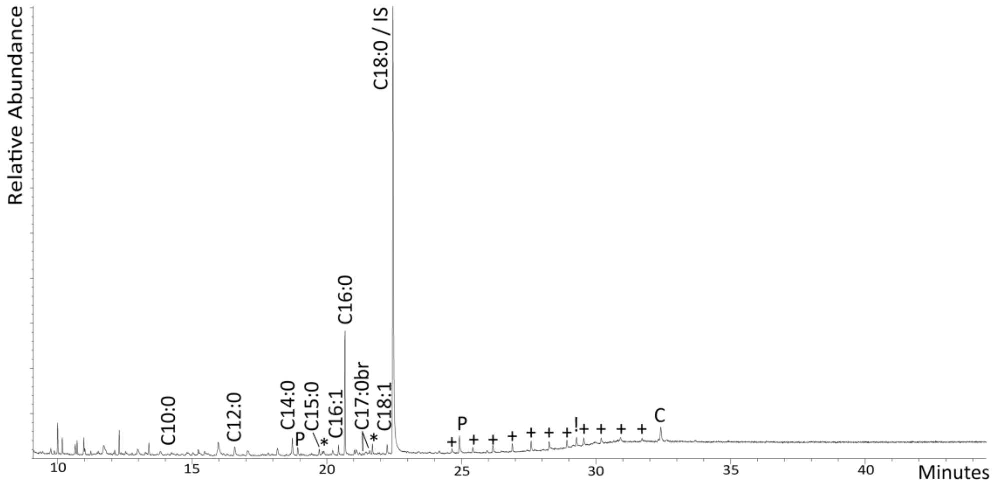

Supplementary Figure S10. Gas Chromatography-Mass Spectrometry results. Total Ion Chromatogram showing the composition of the solvent extracted organic residue adhering to the inner surface of the ilium, run as trimethylsilylated esters. [Cx:y: Fatty acid where x is the carbon number and y is the degree of unsaturation; +: Alkanes (C25-C35); *: Alcohols; !: Cholesterol; P: Phthalate contamination; C: contaminant; IS: Internal standard]

Supplementary Tables

Supplementary Table S1. Description of the experimental tests on three recently extracted lower third molars (M_3) using point tools made on different material.

Specimen ID	Tools	Action	Direction	Inclination	Time
**Ind-1** (RM_3)	bone point	Twisting, clockwise rotation and helicoidal lever	Mesial>distal	50°	45’
**Ind-2** (LM_3)	backed point	Helicoidal lever, twisting, clockwise rotation	Mesial>distal	60°	1’30’
**Ind-3** (LM_3)	wood point	Helicoidal lever and twisting	Mesial>distal	55°	28’

Supplementary Table S2. Description of the experimental tests on six medieval molars.

Specimen ID	Tools	Action	Direction	Inclination	Time
**Vald-1** (RM_3)	Backed point	Clockwise rotation, helicoidal lever and twisting	Mesial>distal	60°	9’25"
**Vald-2** (RM_3)	Backed point	Clockwise/Counterclockwise bidirectional rotation	Mesial>distal	60°	8’21’’
**Guid-1** (RM_2)	Backed point	Helicoidal lever and twisting	Mesial>distal	60°	3’29’’
**Guid-2** (RM_3)	Hafted backed point	Counterclockwise rotation	Lingual>bucco-distal drilling	60°	1’25’’
**Guid-3** (RM^2)	Hafted backed point	Counterclockwise rotation	Mesial>buccal	60°	0.47’
**Guid-4** (LM_2)	Backed point	Inverse and direct (clockwise)	Mesial>buccal	60°	2’27"

Supplementary Table S3. Gas-Chromatography Mass-Spectrometry (GC-MS) results. Table listing the location from where each of the samples were taken and the quantity tested, the corresponding total lipid extract (TLE, Tr. denotes trace amounts), and the lipids identified after GC-MS analysis of the solvent extracted residue. Saponification yielded saponification yielded insignificant quantities of lipid residues results.

Code	Sample	Weight (mg)	TLE (µg)	Lipids identified by GC-MS
**VIL01**	Ilium (inner)	1.4	< 10	**Fatty acids:** $C_{10:0}$, $C_{12:0}$, $C_{14:0}$, $C_{15:0}$, $C_{16:1}$, $C_{16:0}$, $C_{17:0br}$, $C_{18:1}$, $C_{18:0;}$ **Alcohols:** C_{16}, $C_{18;}$ **Alkanes:** C_{25}, C_{26}, C_{27}, C_{28}, C_{29}, C_{30}, C_{31}, C_{32}, C_{33}, C_{34}, C_{35}; **Sterols:** Cholesterol; **Phthalate Plasticiser**
**VIL02**	Ilium (outer)	3.0	Tr.	**Fatty acids:** $C_{12:0}$, $C_{14:0}$, $C_{15:0}$, $C_{16:1}$, $C_{16:0;}$ **Alcohols:** C_{16}, $C_{18;}$ **Alkanes:** C_{24}, C_{25},C_{26}, C_{27}, C_{28}, C_{29}, C_{30}, C_{31}, C_{32}, C_{33}, C_{34}, $C_{35;}$ **Disaccharide; Phthalate Plasticiser**
**VIL03**	Soil control	159.4	Tr.	**Fatty acids:** $C_{16:0}$, $C_{18:0;}$ **Alcohols:** $C_{18;}$ **Phthalate Plasticiser**
**VIL04**	Organic material in concretion	27.2	Tr.	**Fatty acids:** $C_{16:0}$, $C_{18:0;}$ **Alcohols:** $C_{18;}$ **Phthalate Plasticiser**
**VIL05**	Tooth cavity	n/a	Tr.	**Fatty acids:** $C_{14:0}$, $C_{15:0}$, $C_{16:0}$, $C_{18:0;}$ **Alcohols:** C_{18}

Cx:y: Fatty acid where x is the carbon number and y is the degree of unsaturation; Cx: Alcohols where x denotes the carbon number; Br: branched.

Supplementary Table S4. Results of calculus samples and phytolith identification.

Sample	Results
Cavity rinse	10 starches, all polyhedral with centric fissured hila. Several fibres and chunky material, no phytoliths
Stuffing from mandible box	49 starches, all polyhedral with centric fissured hila. Several are damaged (cracked, broken, etc.)
Bubble wrap from mandible box	7 starches, polyhedral with centric fissured hila, many fibres, one pollen grain probably from pine
Fragments from skull box	Many fibres, particles, and chunks, no starch, no phytoliths
Stuffing from skull box	12 starches, polyhedral with centric fissured hila, many fibres, some with starches attached
Day 1 control slide	Very clean slide, a few fibres
Day 2 control slide	Clean slide, a few fibres, one plant particle with bordered pits
Day 3 control slide	Very clean slide, a few fibres

Supplementary Video Legends

Supplementary Video 1. Results obtained using the Occlusal Fingerprint Analyser (OFA) software for the right M3 pair. Virtual results showing detected sequential antagonistic occlusal contacts covering the wear facet pattern on the right M3s. The dominant horizontal occlusal direction is protrusive. Green highlights previous and red current contacts.

Supplementary Video 2. Results obtained using the Occlusal Fingerprint Analyser (OFA) software for the complete Villabruna dentition. Virtual models of the reconstructed Villabruna specimen dental arches after simulating a bite situation on the right side. Sequential occlusal contacts are visible on the lower arch model. Green highlights previous and red current contacts.
